# Effect of altitude zone exposure on visuospatial function in military aircrew member

**DOI:** 10.11604/pamj.2022.41.235.22274

**Published:** 2022-03-22

**Authors:** Nanda Mardas Saputra, Indah Suci Widyahening, Herman Mulijadi, Widura Imam Mustopo, Retno Asti Werdhani, Nurhadi Ibrahim, Retno Wibawanti

**Affiliations:** 1 Department of Community Medicine, Faculty of Medicine Universitas Indonesia, Jakarta, Indonesia,; 2 Department of Physiology, Faculty of Medicine Universitas Indonesia, Jakata, Indonesia

**Keywords:** Visuospatial, hypoxia, aviation, altitude, performance, cognitive, aircrew

## Abstract

**Introduction:**

visuospatial is a type of high-level visual perception necessary for identification, information integration, analysing of visual and spatial forms, details, structures and spatial relation. These functions are required in order to successfully complete aviation-related tasks, such as analysing movement, distance perception, and spatial navigation. The aim of this study is to examine whether hypobaric hypoxia can significantly influence changes in visuospatial function, thus increasing the risk of accident or serious incident during flight operation.

**Methods:**

this study is a quasi-experiment of pre-post study including before and after hypobaric hypoxia simulated through an altitude chamber. In this study, 42 military aircrews were exposed to different altitude zones at ground level, 10,000 ft (ft) and 25,000 ft respectively, for five minutes. At each altitude zone, the participants were instructed to complete a clock drawing test as a measurement for visuospatial function. The results were analysed using the McNemar non-parametric test.

**Results:**

among the 42 subjects, six show impaired visuospatial function at 10,000 ft and 26 participants show it at 25,000 ft. There were significant increased on the proportion of impaired visuospatial function between the ground level to 10,000 ft (p=0.031), 10,000 to 25,000 ft (p=0.0001) and ground level to 25,000 ft (p=0.0001).

**Conclusion:**

hypobaric hypoxia may have a significant influence on visuospatial function, starting from as early as 10,000 ft to 25,000 ft. This decrease of visuospatial function could affect human cognitive performance when flying and increase the risk of aviation accidents.

## Introduction

Humans, like many other terrestrial animals, evolved to live on the earth´s surface. Significant technological advances allow humans to traverse the vast sky, and even explore beyond it. This achievement is possible, not because we as a humans were able to conquer and bend nature but rather because we obey nature and understand how it works in association with human performance and physiology [1].

The success and safety of a flight are determined by many factors that interact with each other dynamically. This was said by Edwards in 1972 who proposed the concept of Software, Hardware, Environment and Liveware (SHELL) [2]. Variability in environment is a challenge that must be understood by those in the field of aviation. This Variability can introduce hazards such as atmospheric pressure, acceleration exposure, vibration, thermal and radiation exposure. Altitude reflected by atmospheric pressure is a specific hazard in aviation environments, whereas different altitude zones such as sea level, physiological efficient zone (10,000 ft) and the physiological deficient zone (25,000 ft), have their own impact and influence on human function and physiology [3].

Hypoxia is a hazard specific to aviation, and is generally understood that an increase in altitude will result in the progression of hypoxia and its symptoms. Based on empirical studies, it is generally accepted that flying higher than 10,000 ft (3048 m) above sea level requires adequate supplemental oxygen as a countermeasure for hypoxia and its symptoms [1]. Performance on previously learned coding and conceptual reasoning tasks is unaffected up to approximately 10,000 ft. However, short term and long term memory are affected significantly as early as 8,000 ft and worsen as altitude increased [3]. This could influence aircrew on novel tasks. Similarly, another study showed that there is a significant effect on the rate of learning at 8,000 ft, suggesting that hypoxia affected learning and memory at lower altitudes than 10,000 ft [4]. Impairment in codification and short-term memory are noticeable above altitude of 6,000 m. Alterations in accuracy and motor speed are identified at lower altitudes. Deficits in verbal fluency, language production, cognitive fluency, and metamemory are also detected [5].

However, a study by Pearson and Neal (1970) showed that hypoxia caused by exposure to an altitude of 8,000 - 10,000 ft has no detectable effect on performance if the task was well-learned first at ground level [6]. A series of studies by Kelman and Crow (1971) also showed that memory and learning were not affected by hypoxia at any altitude below 12,000 ft [7,8]. In a more recent study by Pilmanis, the author found that exposure to low-grade hypoxia (5,000 - 12,000 ft) has minimal influence on cognitive performance in contrast to some existing symptoms associated with hypoxia [9].

A visuospatial function can be simply defined as a perception or thought process that involves knowledge and awareness of a visual and spatial phenomenon. In more complex terms visuospatial perception is a cognitive process required for identifying, integrating and analysing visual/spatial forms, details, structures and the spatial relationship of two to three-dimensional forms. Visuospatial ability or function is needed particularly for analysing movement and distance, shape perception, spatial navigation, as well as perceiving and reconstructing mental image from two dimensions into three, all of which are important for aviation-related tasks [10,11]. Visuospatial function relies on various neurocognitive abilities, including fine motor coordination, comprehension of visual-spatial relations, planning decision making and executive function [12].

Hypobaric hypoxia, that is, hypoxia caused by changes in atmospheric pressure, can cause the physiological effects to the human body, particularly in neurocognitive function. The purpose of this study is to examine whether hypobaric hypoxia can significantly influence changes in visuospatial function, thus increasing the risk of accident or serious incident during flight operation. As it has been shown by the previous studies regarding cognitive performance we hypothesized that visuospatial function would be impaired by the increase of altitude.

## Methods

This study used an experimental one-group pretest-posttest design and conducted at the Indonesian Air Force Institute of Aviation Medicine, Lakespra (Lembaga Kesehatan Penerbangan) Dr Saryanto, Jakarta. The study has been reviewed and approved by The Research Ethics Committee of the Faculty of Medicine Universitas Indonesia - Cipto Mangunkusumo Hospital number KET-497/UN2.F1/ETIK/PPM.00.02/2019. The subjects included in this study were healthy male military aircrew members participated in an Aerophysiology Indoctrination Training between May to June 2019, all had carried out a medical examination before entering the hypobaric chamber and were willing to take part in the study and signed the informed consent form. Those with sinus disturbances, had dental carries with impaired filling or contagious respiratory tract infection were excluded. In determining the sample size we used two-group repeated-measures experiments sample size calculation and found the minimum sample size of 34 was required [13], we decided to include 42 subjects to ensure the result of our study.

The independent variable of this study is the altitude zone, while the dependent variable is the visuospatial function. The altitude zone refers to the altitude zone´s effect on the body. All subjects were instructed to perform the clock drawing test (CDT) with Shulman scoring [14-16] to measure visuospatial function at three different levels of altitude simulating the altitude zones at ground level, 10,000 ft and 25,000 ft, in a hypobaric chamber. The simulation started from the ground to 5,000 ft for a sinus check before descending back to ground level. Subjects were then elevated to a simulated 10,000 ft and instructed to remove their oxygen masks before performing the CDT for approximately five minutes. Visuospatial function was measured using clock drawing test which was conducted approximately three minutes after subjects were instructed to remove the mask. Instructions were given from outside the chamber using a microphone. The clock drawn by subjects must be presented with three elements which are the circle, number and the hands (minute and hour). Any incompleteness or deviated form of the clock elements were considered as impaired visuospatial function. Subjects that did not draw any form of clock at all were excluded from data collection.

Subjects then instructed to wear the oxygen mask before ascending further to 25,000 ft and instructed to remove the mask again before performing the test again for 5 minutes. While performing the test, subjects were closely observed and monitored for both objective and subjective symptoms of hypoxia. To ensure the safety of the participants, any subject showing severe symptoms or not able to continue the test further will be instructed to wear the mask immediately and to be moved to a separate chamber where medical emergency team with appropriate equipment was on standby to check and perform emergency treatment. After finishing the test, subjects wore their oxygen masks before descending back to ground level.

## Results

Between 8 May to 26 June 2019 there were 95 military aircrews attended the Aerophysiology Indoctrination Training at the Indonesian Air Force Institute of Aviation Medicine, Lakespra (Lembaga Kesehatan Penerbangan) Dr Saryanto, Jakarta. Out of 95 subjects, 42 are eligible for the study using the inclusion and exclusion criteria. Characteristics of the 42 male subjects who meet the eligibility criteria is presented in Table 1.

**Table 1:** characteristic of the military aircrews (n=42)

Characteristics	Median (Max-Min)	n (%)
Age	28.00 (19-54)	
Type of flights		
Fixed wing - Combat		13 (31%)
Fixed wing - Transport		18 (42.9%)
Helicopter		11 (26.2%)
Job		
Pilot		23 (54.8%)
Flight engineer		18 (42.9%)
Air Navigator		1 (2.4%)
Total flying hours	200 (0-10,000)	
Time of useful consciousness (TUC)	254.2 ±51.9*	

The effect of hypoxia on visuospatial function started to present significantly at 10,000 ft, and the number of subjects with impaired visuospatial function increased more as the altitude increased to 25,000 as shown in Table 2. there was significant change in the visuospatial function between ground level and 10.000 ft and between ground level and 25.000 ft with p value 0.031 and 0.0001 respectively (McNemar test, p<0.05). Subjects reported a range of typical symptoms of hypoxia during the test such as headache, palpitation, abdominal discomfort or ear pain however, no subjects reported any lasting or permanent effects after the test.

**Table 2:** changes in the visuospatial function of the military aircrews in different altitude (n=42)

Altitude	Normal		Impaired		P (McNemar)
	n	%	n	%	
Ground Level	42	(100)	0	(0)	
Physiological efficient zone (10,000 ft)	36	(85.7)	6	(14.3)	0.031
Physiological deficient zone (25,000 ft)	16	(38.1)	26	(62.9)	0.0001

Some subjects with impaired visuospatial function can only draw the circle with incomplete numbers and no hands, others showed partial drawing and tight grouping of numbers and hands or changes of the size of the clock that became smaller and difficulty to read correctly.

## Discussion

This study has shown that at 10,000 ft (the physiological efficient zone) and 25,000 ft (the physiological deficient zone) there is a significant increase in the proportion of subjects with impaired visuospatial function. However, the variability in finding regarding the effect of hypobaric hypoxia on human performance at an altitude 10,000 ft from different studies is something that should be noted. Visuospatial functioning is one of many aspects of the whole neurocognitive function in humans. Most research agreed that by an elevation of 10,000 ft, significant changes in short-term and long-term memory start to become noticeable. The findings of this study support that there is a significant impact on human cognitive performance at an altitude of at least 10,000 ft perhaps lower. Other factors that may cause the measured variation in visuospatial function related to hypoxia are fatigue, smoking, and physical fitness. These factors can affect cardiorespiratory function, which can lead to lower tolerance against hypoxic environments. Although a high proportion of visuospatial impairment was expected at 25,000 ft, participants were only asked to spend short periods of time at this simulated elevation level and did not report any lasting symptoms or issues after returning to ground level.

Several mechanisms and pathophysiologic processes are likely involved in the alteration of visuospatial function. The most likely factor is changes in the metabolic process, from aerobic to anaerobic, caused by low levels of oxygen needed in TCA (tricarboxylic acid cycle). Low Adenosine Triphosphate (ATP) production will impair the function of Na^+^/K^+^-ATPase which causes accumulation of intracellular sodium. This increase of intracellular sodium then causes a passive influx of water and Cl- into the cell, leads to cellular swelling and then to cellular dysfunction. AMPA (α-amino-3-hydroxy-5-methyl-4-isoxazolepropionic acid) receptors of glutamate function are impaired and unable to make significant EPSP (excitatory postsynaptic potential) needed to open Mg+ blockade on the NMDA (N-methyl-D-aspartate) channel and prevent the influx of Ca+ ions for creating long term potentiation for synaptic plasticity [17-19]. As stated before, no subjects reported any permanent symptoms after exposure, which means that it is unlikely any process involving cellular or neuronal damage, but to confirm this would require additional data. Certain limitation in this study is we cannot give repetitive exposure to subjects to see whether repeated exposure will cause a certain adaptation or a different outcome.

Further studies could examine potential effects at a lower altitude, such as 5,000 to 8,000 ft, as it is the typical range of standard pressure of pressurized cabins in aircrafts. The effect of repetitive exposure or intermittent hypoxia should be investigated as well. A high number of aviation-related accidents in west Papua share particular characteristics, not only that the terrain is already high, but typically flights are of relatively short distances therefore in one day there could be many flights. This, combined with factors such as possible fatigue due to the high number of take-offs and landings per day, may contribute to the high number of aviation accidents in west Papua. The findings from this study should increase the awareness on the importance of adequate oxygen supply and its equipment especially for unpressurized cabin aircraft flying on high terrain area and to better educate aircrew on the effect of hypobaric hypoxia against human cognitive performance and its impact against the safety of flight operation.

## Conclusion

Our results show that visuospatial function is impaired significantly at level of 10,000 ft and 25,000 ft. As visuospatial function relies on memory function and perception as part of overall neurocognitive function, physiological changes of synaptic plasticity and neurotransmitter function due to low level of oxygen are the mechanisms responsible for the decrease of visuospatial function. This finding support the risk of flying on unpressurized cabin aircraft without adequate oxygen supply particularly at 10,000 ft and this altitude should only be reserved for emergency rather than for normal cruising altitude.

### What is known about this topic

- *Visuospatial function as a part of the whole neurocognitive function is an important aspect of the human factor in aviation;*
- *The increase of altitude reflected by the decrease of atmospheric pressure altered many basic human functions and its physiology;*
- *10,000 ft is the common safe borderline where a human can function properly without any supplemental oxygen*.

### What this study adds

- *There is evidence that as low as 10.000 ft there is already a significant change in human function particularly in visuospatial function*.
